# Netrin‐1 Promotes Inflammation Resolution to Achieve Endothelialization of Small‐Diameter Tissue Engineering Blood Vessels by Improving Endothelial Progenitor Cells Function In Situ

**DOI:** 10.1002/advs.201700278

**Published:** 2017-09-28

**Authors:** Yanzhao Li, Simin Wan, Ge Liu, Wang Cai, Da Huo, Gang Li, Mingcan Yang, Yuxin Wang, Ge Guan, Ning Ding, Feila Liu, Wen Zeng, Chuhong Zhu

**Affiliations:** ^1^ Department of Anatomy National and Regional Engineering Laboratory of Tissue Engineering State and Local Joint Engineering Laboratory for Vascular Implants Key Lab for Biomechanics and Tissue Engineering of Chongqing State Key Laboratory of Trauma, burn and Combined injury Third Military Medical University Chongqing 400038 China

**Keywords:** endothelial progenitor cells, exosomes, inflammation resolution, macrophage reprogramming, small‐diameter tissue engineering blood vessels

## Abstract

The transplant of small‐diameter tissue engineering blood vessels (small‐diameter TEBVs) (<6 mm) in vascular replacement therapy often fails because of early onset thrombosis and long‐standing chronic inflammation. The specific inflammation state involved in small‐diameter TEBVs transplants remains unclear, and whether promoting inflammation resolution would be useful for small‐diameter TEBVs therapy need study. The neural protuberant orientation factor 1 (Netrin‐1) is found present in endothelial cells of natural blood vessels and has anti‐inflammatory effects. This work generates netrin‐1‐modified small‐diameter TEBVs by using layer‐by‐layer self‐assembly to resolve the inflammation. The results show that netrin‐1 reprograms macrophages (MΦ) to assume an anti‐inflammatory phenotype and promotes the infiltration and subsequent efflux of MΦ from inflamed sites over time, which improves the local microenvironment and the function of early homing endothelial progenitor cells (EPCs). Small‐diameter TEBVs modified by netrin‐1 achieve endothelialization after 30 d and retain patency at 14 months. These findings suggest that promoting the resolution of inflammation in time is necessary to induce endothelialization of small‐diameter TEBVs and prevent early thrombosis and problems associated with chronic inflammation. Furthermore, this work finds that the MΦ‐derived exosomes can target and regulate EPCs, which may serve as a useful treatment for other inflammatory diseases.

## 1 Introduction

Cardiovascular disease is a leading cause of death that creates a significant burden on patients and requires replacement therapy to treat the damaged vessels. Small‐diameter tissue engineering blood vessels (TEBVs) have been used with increasing frequency for vascular replacement therapy, and the construction of small‐diameter TEBVs that undergo endothelialization in vivo (without endothelial cells, ECs, seeded in vitro) has been at the forefront of the field.^1^ However, the grafts have been leading to high incidences of thrombosis, intimal hyperplasia, and calcification by chronic graft rejection due to innate immune responses and chronic inflammation,^2^, ^3^ which has become the primary challenge for the clinical applications of small‐diameter (1.5–2 mm or less) TEBVs.^4^, ^5^

Recent studies have found that acute inflammation initiates the regenerative response, and resolution by macrophages (MΦ) is necessary for survival of regenerative cells.^6^, ^7^ For example, CD86‐MΦ induces high expression of proinflammatory cytokines and reactive oxygen species, whereas CD163‐MΦ induces high expression of scavenging molecules and anti‐inflammatory cytokines and promotes tissue remodeling.^8^ Exosomes released by MΦ or other inflammatory cells represent a new component of the inflammatory microenvironment that play an important role in the cell's ability to communicate with its environment.^9^, ^10^, ^11^, ^12^

Endothelial progenitor cells (EPCs) are precursor stem cells of ECs that can be mobilized from the bone marrow and targeted to specific sites to promote angiogenesis and early endothelialization of small‐diameter TEBVs.^13^, ^14^ Studies have shown that reduced inflammatory response can promote mobilization and targeting of early EPCs.^15^, ^16^ However, excessive or sustained inflammation can reduce the presence of EPCs in the blood and cause problems such as thrombosis and intimal hyperplasia.^17^ Therefore, it is critical to promote inflammation resolution of small‐diameter TEBVs and provide a favorable microenvironment for the growth and differentiation of EPCs into ECs.

Previous studies have found that netrin‐1 is present in the ECs of natural blood vessels and can promote angiogenesis through uncoordinated‐5 homolog B (UNC5B) and deleted in colorectal cancer (DCC) receptors.^18^, ^19^, ^20^ Additionally, netrin‐1 prevents ischemia and exerts anti‐inflammatory effects by binding to the adenosine receptor A2b.^21^

In the present study, we hypothesized that netrin‐1 could reprogram MΦ to exert anti‐inflammatory effects and promote inflammation resolution to establish a favorable microenvironment for EPCs, thereby achieving rapid endothelialization and improving long‐term patency rate of small‐diameter TEBVs.

After testing and verifying our hypothesis and the underlying mechanism in vitro, we constructed the small‐diameter TEBVs from decellularized rat arteries and modified them for the controlled release of netrin‐1 by chitosan. The modified small‐diameter TEBVs were then transplanted into the carotid arteries of rats for subsequent evaluations of patency and endothelialization, and the relationships between these parameters were examined.

## 2 Results

### 2.1 Netrin‐1 Reprograms MΦ into CD163 via the A2b Receptor In Vitro

**Figure 1:**
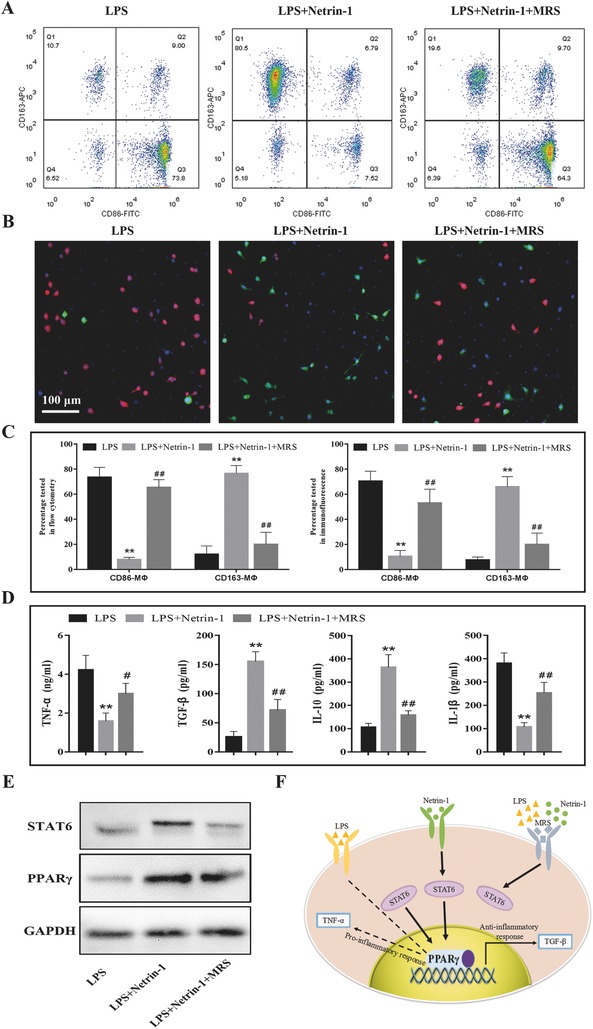
Netrin‐1 reprograms MΦ to express CD163 by enhancing mitochondrial energy metabolism. A) Flow cytometry analysis showed the cell surface expression of the CD86 and CD163. B) Immunofluorescence staining of the peritoneal MΦ. CD86 and CD163 are stained with red and green fluorophore, respectively; and nuclei are stained with 4′,6‐diamidino‐2‐phenylindole (DAPI). C) Statistical results of CD86‐ or CD163‐positive MΦ. The percentage of two phenotypes of MΦ was tested and analyzed by flow cytometry and immunofluorescence. D) Release of cytokines was analyzed in cell culture media according to each group using enzyme linked immunosorbent assay (ELISA). E) Western blot analysis of PPARγ and STAT6 expression in MΦ treated with LPS or netrin‐1. F) Model for regulation of MΦ reprogramming by LPS to support proinflammatory CD86‐MΦ or netrin‐1 to activate STAT6 and PPARγ to promote expression of anti‐inflammatory CD163‐MΦ. All data are representative of ten independent experiments. Where applicable, results are expressed as mean ± SEM, *p < 0.05, **p < 0.01 versus LPS, ##*p* < 0.01 versus LPS+Netrin‐1 (*n* = 10).

After being stimulated by lipopolysaccharide (LPS) in vitro, flow cytometry analysis showed that the rat peritoneal MΦ can be transformed into proinflammatory MΦ, as demonstrated by the strong expression of CD86, a costimulatory receptor necessary for T cell activation.^22^ MΦ were reprogrammed to be anti‐inflammatory with 250 ng mL^−1^ netrin‐1 and high expression of CD163, a scavenger receptor involved in endocytosis of hemoglobin/haptoglobin, and the transformation rate was up to 69.8%. However, the effect of netrin‐1 was suppressed after the addition of 100 × 10^−6^ m MRS, an A2b receptor antagonist, at an inhibition rate of 60.9% (**Figure** **1**A,C). Immunofluorescence analysis showed that the two types of MΦ had no significant difference in morphology; however, the inflammatory stimulation was regulated by netrin‐1, the transformation rate of CD86‐MΦ changed from 70.5% to 10.5%, and that of CD163‐MΦ changed from 7.5% to 66.0%. MRS inhibited these effects (Figure 1B,C). In addition, we found 250 ng mL^−1^ was the appropriate concentration for netrin‐1 in MΦ reprogramming experiments, and higher concentration had no significant influence on reprogramming efficiency (Figure S1, Supporting Information).

To determine whether cytokine production of MΦ is associated with polarization change, the expression of interleukin‐1β (IL‐1β), tumor necrosis factor‐α (TNF‐α), transforming growth factor‐β (TGF‐β), and IL‐10 was quantified in supernatants of MΦ from each group. Consistent with the proinflammatory and anti‐inflammatory phenotypes of CD86‐MΦ and CD163‐MΦ, our results showed that netrin‐1 suppressed the LPS‐induced increase in IL‐1β and TNF‐α levels in vitro. In contrast, secretion of immunomodulatory TGF‐β and the anti‐inflammatory cytokine IL‐10 was markedly increased in CD163‐MΦ compared with that in CD86‐MΦ (Figure 1D). Similar differences in supernatant levels in CD86‐ and CD163‐MΦ have previously been reported.^23^ Our results also showed that the anti‐inflammatory effect of netrin‐1 could be blocked by MRS.

The signaling pathway through which netrin‐1 suppresses inflammation is not known. It was previously reported that peroxisome proliferator‐activated receptor (PPAR) pathways might be primarily involved owing to their known role in MΦ polarization.^24^ We tested PPARγ and STAT6 expression by western blot analysis and found the levels of both proteins were increased following netrin‐1 treatment and decreased following MRS treatment (Figure 1E).The possible regulatory pathways of LPS and netrin‐1 is shown in the schematic diagram (Figure 1F).

### 2.2 MΦ‐Derived Exosomes Are Actively Incorporated by EPCs In Vitro and In Vivo

**Figure 2:**
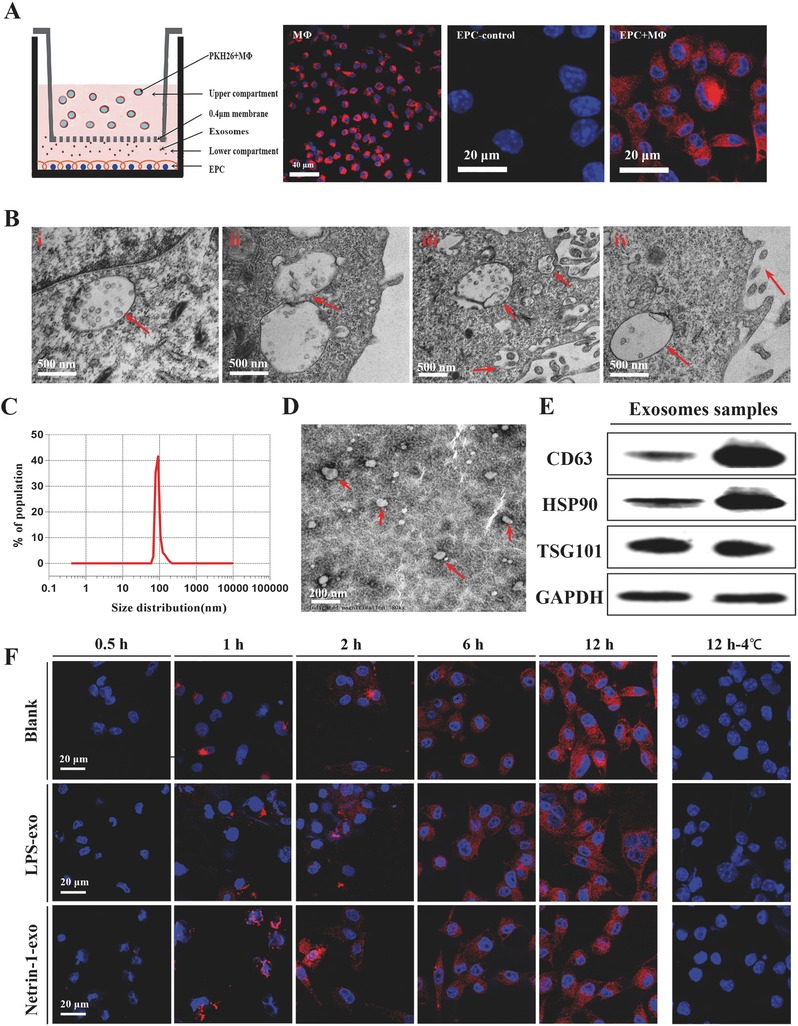
MΦ secrete exosomes that enter endothelial progenitor cells in culture; A) A 24 h coculture assay of MΦ and EPCs at 37 °C. PKH‐26 stained MΦ were cultured in upper compartment, EPCs were adherent cultured in the lower compartment and exosomes were transferred through the 0.4 µm. Representative image of *n* = 10 experiments. B) Transmission electron micrograph (TEM) of MΦ secreting exosomes i) cytoplasm with MVBs enclosing numerous bilipidic layer‐bound exosomes (red arrows), ii) inward invagination (red arrows) in the MVB membrane demonstrates the beginning of exosomes biogenesis, iii) MVB fusing with cytomembrane, and iv) exosomes are secreted out from the macrophage. C) Size distribution measurement of isolated exosome population demonstrates a single peak at nearly 100 nm and free contamination. D) TEM analyzed MΦ‐derived exosomes. The image shows small vesicles of ≈100 nm in diameter. E) Western blotting reveals expression of the MΦ exosome markers CD63, HSP90, and TSG101. F) In the coculture assay at 37 °C, MΦ‐derived exosomes were taken up by EPCs in each group. Images were taken up at various time intervals 0.5, 1, 2, 6, and 12 h. In the coculture assay at 4 °C, images were taken up at 12 h.

We observed a transfer of membrane vesicles from MΦ to EPCs based on the coculture of PKH26‐labeled primary MΦ with EPCs for 24 h (**Figure** **2**A). Several multivesicular bodies (MVBs) were identified in the cytoplasm of MΦ by transmission electron microscopy (TEM), carrying bilipid membrane‐bound exosome‐like vesicles. The MVB membranes then invaginated and initiated the biogenesis of exosomes as previously shown,^25^ MVBs fused to the cell membrane and released the exosome‐like vesicles to the extracellular space (Figure 2B). The morphology and phenotypes of isolated vesicles were identified as described previously.^26^ First, we found that the vesicle diameter was 108 nm on average and ranged from 50 to 150 nm (Figure 2C). Second, the morphology of the MΦ‐derived vesicles was observed by TEM, and the vesicles were found to be round in shape with a diameter of ≈100 nm (Figure 2D). Finally, the exosome marker proteins CD63, HSP90, and TSG101 were detected in the MΦ‐derived exosomes (Figure 2E). These properties suggest that the transferred membrane vesicles from MΦ to EPCs in our experiments were indeed exosomes.

From the in vitro transwell experiment, we found that exosomes were taken up by MΦ 1–2 h after application and accumulated in EPCs over time. There was no significant difference in exosome number and uptake time between groups. Exosome uptake was impaired after incubation at 4 °C, which confirmed that exosome uptake was mediated by a biologically active process (Figure 2F). The in vivo experiment revealed the accumulation of exosomes in EPCs of the spleen and peripheral blood (PBL) within 24 h after injection; 5.06% PBL EPCs and 4.89% spleen EPCs had phagocytosed labeled exosomes (Figure S2, Supporting Information).

### 2.3 Reprogrammed MΦ Transfer Functional lncRNAs to EPCs through Exosomes, Altering the Migration, Proliferation, and Tube Formation of EPCs

**Figure 3:**
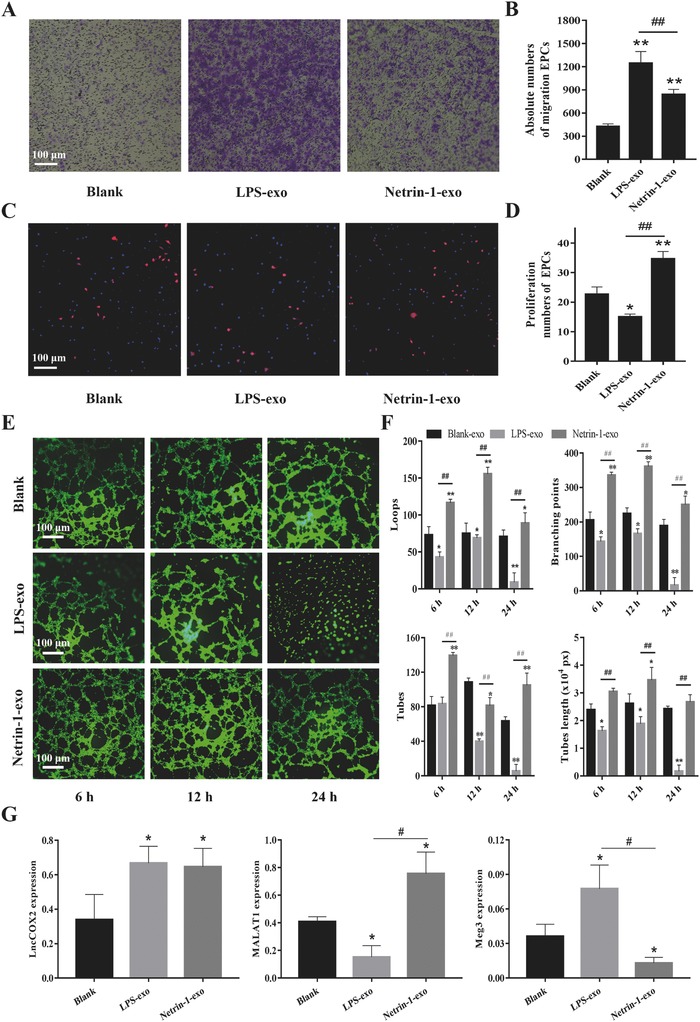
Exosomes secreted by netrin‐1‐modified MΦ affect EPCs migration, proliferation, and tube formation in vitro. A) The exosomes influenced the migration of EPCs. The medium in the lower chamber contained 100 µg mL^−1^ exosomes derived from LPS or netrin‐1‐stimulated MΦ or blank control in the transwell assay. B) The absolute number of EPCs migrating to the lower chamber in each group. C) DAPI (blue) was used to stain nuclei of all cells and EdU (red) was incorporated into EPCs that were proliferating in each group. D) The percentage of actively proliferating EPCs in each group. E) EPCs were cultured in 96‐well plates previously coated with Matrigel and incubated in each group. Images were taken at 6, 12, and 24 h. F) Several parameters of the tube formation assay including loops, branching points, tubes and tubes length were quantificated using ImageJ. G) Relative expression level of Tie‐1ASlncRNA, lnc‐Ang362, lncRNA‐MALAT1, and lncRNA‐Cox2 in exosomes of MΦ in each group. Total RNA was extracted from exosomes and quantitative real time polymerase chain reaction (qRT‐PCR) was performed. Representative image of *n* = 10 experiments. **p < 0.01 versus control, ##*p* < 0.01 Netrin‐1‐exo versus LPS‐exo.

Exosomes are useful for regulating cell–cell communication and we have found MΦ‐derived exosomes that enter EPCs in vitro and in vivo. We then pretreated the EPCs with MΦ‐derived exosomes of different phenotypes to determine whether the EPCs specific effects on inflammation were caused by these exosomes. EPCs migration is a critical initiating event for rapid endothelialization.^15^ Using a Transwell migration assay, we found that the number of migrated cells increased 0.92–1.14‐fold following netrin‐1 treatment compared to the control, whereas the number of migrated cells treated with LPS significantly increased 1.82–2.58‐fold compared to the control, and 0.35–0.62‐fold compared to the cells treated with netrin‐1 (**Figure** **3**A,B). EdU incorporation, reflecting EPCs proliferation, significantly increased by 1.46–2.2‐fold following netrin‐1 treatment compared to the control, whereas cell proliferation decreased by 0.4–0.7‐fold following LPS treatment (Figure 3C,D). Netrin‐1 promoted exosome‐induced EPCs tube formation compared to the control, whereas LPS inhibited tube formation at all time intervals and most notably at 24 h (Figure 3E). We evaluated vessel parameters including the following: the number of closed loops, branching points, the tubes, and the total length of the tubes. The results showed they all decreased significantly in the LPS group especially at 24 h and increased in the netrin‐1 group. The tubes number and length of tubes was also strong decreased at 12 h in the LPS group, and netrin‐1‐exo obviously inhibited the decrease (Figure 3F).

To identify lncRNA that might control EPCs function, we measured the expression of several angiogenesis related lncRNA (Tie‐1ASlncRNA, lnc‐Ang362, lnc‐Cox2, MALAT1, Meg3, and Long Noncoding RNA (MANTIS)) in exosomes derived from MΦ. The results revealed that several of them were statistically different, Meg3 expression decreased in CD163‐MΦ‐derived exosomes, and increased in CD86‐MΦ‐derived exosomes. MALAT1 expression increased in CD163‐MΦ‐derived exosomes and decreased in CD86‐MΦ. LncRNA‐Cox2 expression increased in CD86‐MΦ‐derived exosomes and remained unchanged in CD163‐MΦ‐derived exosomes (Figure 3G).

### 2.4 Construction and Characterization of the Nanostructured Small‐Diameter TEBVs by Netrin‐1

**Figure 4:**
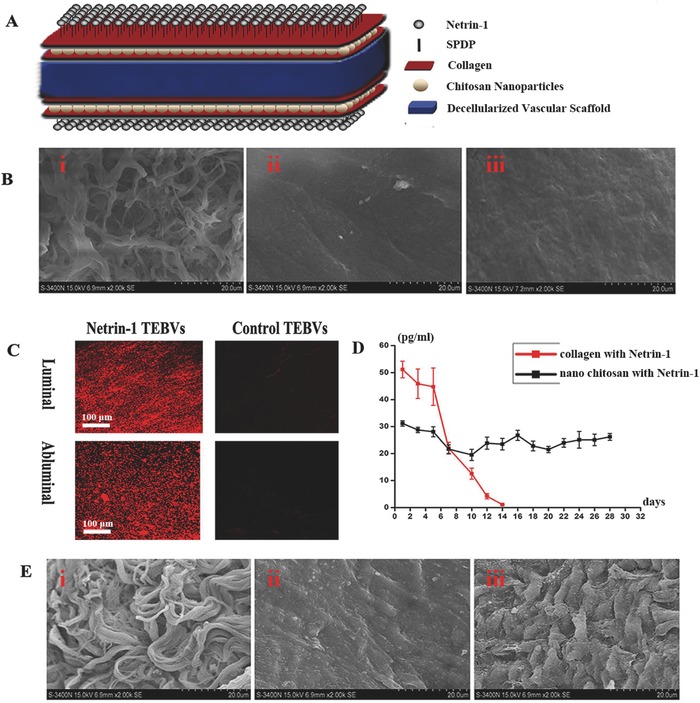
Layer‐by‐layer self‐assembly of small‐diameter TEBVs resists blood flow washing. A) Layer‐by‐layer self‐assembly was used to modify the decellularized vascular scaffold. B) SEM image of the luminal surface of the vessel scaffold after the decellularization process i), the surface modification of the first layer of collagen ii), and the surface modification of chitosan nanoparticles in the second layer of collagen iii). C) The luminal surface and the abluminal surface of the netrin‐1 and control small‐diameter TEBVs were immunostained for netrin‐1 (red). D) Controlled release of netrin‐1 from chitosan nanoparticles localized at the scaffolds for 4 weeks. E) SEM image of the luminal surface of small‐diameter TEBVs cross‐linked with only a layer of collagen i), control small‐diameter TEBVs without netrin‐1 modification ii), and netrin‐1‐modified small‐diameter TEBVs iii) at 1 month after transplantation.

To study the effect of netrin‐1 on patency of small‐diameter TEBVs, we employed a rat model and transplanted the small‐diameter TEBVs into rats. The schematic diagram shows the layer‐by‐layer self‐assembly modified netrin‐1 both existed on the surface and inside the chitosan nanoparticles in small‐diameter TEBVs (**Figure** **4**A). The interaction between the carboxylic acid of collagen and the amine of chitosan creates an ionic complex and improves stability and strength (Figure S3A, Supporting Information).^27^ Scanning electron microscopy (SEM) was used to visualize the luminal surface of the vessel scaffold during the modification process from decellularization to deposition of collagen and incubation of chitosan nanoparticles on small‐diameter TEBVs (Figure 4B). The immunofluorescence result showed *N*‐succinimidyl 3‐(2‐pyridyldithio) propionate successfully couple netrin‐1 to the collagen surface both inside and outside of the vessel scaffold based on the receptor‐ligand interaction, and small‐diameter TEBVs in control group that only crosslinked two layers of collagen and modified without netrin‐1 had no fluorescence (Figure 4C and Figure S3B, Supporting Information). In vitro assessments indicated that the chitosan nanoparticles released netrin‐1 at a consistent rate (24.82 pg mL^−1^ per day) over 30 d (Figure 4D); however, netrin‐1 could no longer be detected at 60 d. The surface materials in the modified small‐diameter TEBVs, which were crosslinked only to a layer of collagen on the decellularized arteries, had been washed away by blood flow at 1 month after implantation. The control small‐diameter TEBVs resisted blood flow but the cells failed to attach to them, whereas the netrin‐1 modified small‐diameter TEBVs resisted blood flow and promoted endothelialization 1 month after implantation (Figure 4E).

### 2.5 Netrin‐1 Reprograms MΦ Infiltrated in Small‐Diameter TEBVs and Promote Inflammation Resolution In Vivo

**Figure 5:**
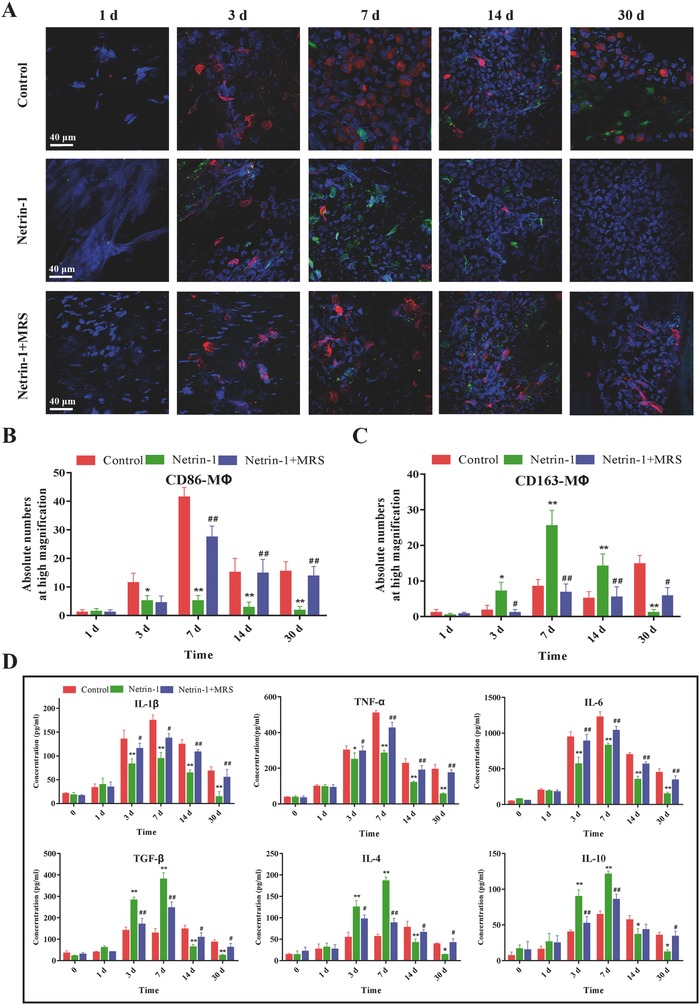
Netrin‐1 reprograms MΦ infiltrated in small‐diameter TEBVs and promotes inflammation resolution in vivo. A) Immunofluorescence staining of CD86 (red) and CD163 (green) colocalized with DAPI (blue) on the intima in the small‐diameter TEBVs in each group at various time intervals 1, 3, 7, 14, and 30 d after implantation. B) Quantitative comparison of the absolute numbers of CD86‐MΦ at high magnification in the control, Netrin‐1, and Netrin‐1+MRS groups. C) Quantitative comparison of the absolute numbers of CD163‐MΦ at high magnification in each group. D) ELISA showed that netrin‐1 significantly decreased the levels of all of the proinflammatory cytokines IL‐1β, TNF‐α, and IL‐6 and increased the anti‐inflammatory cytokine TGF‐β, IL‐4, and IL‐10. MRS inhibited the effects of netrin‐1. All data are representative of ten independent experiments. Results are expressed as mean ± SE, *p < 0.05, **p < 0.01 versus control, ##*p* < 0.01 versus Netrin‐1.

Representative confocal images revealed the presence of CD86‐MΦ and CD163‐MΦ infiltrated on the intimal layer of small‐diameter TEBVs in control, netrin‐1, and netrin‐1 + MRS groups. We found that MΦ were infiltrated from 1 to 3 d after implantation and accumulated on intima over time, peaking after 7 d in three different groups. MΦ concentration decreased from 7 to 14 d and nearly disappeared at 30 d in the group treated with netrin‐1, whereas MΦ (especially CD86‐MΦ) were still present in the control and netrin‐1 + MRS groups (**Figure** **5**A). Further quantitative analysis showed that the netrin‐1 group had the lowest amount of CD86‐MΦ and the greatest amount of CD163‐MΦ after 3, 7, and 14 d compared with the other two groups (Figure 5B,C), indicating that reprogramming and phenotype transformation of MΦ occurred in the netrin‐1 group. Conversely, in the control and netrin‐1 + MRS groups, CD86‐MΦ were widespread and significantly more numerous than in the netrin‐1 group, especially at 3 and 7 d after implantation.

The expression patterns of three proinflammatory cytokines IL‐1β, TNF‐α, and IL‐6 were all reduced in cells treated with netrin‐1 at 3, 7, 14, and 30 d, and the expression patterns of three anti‐inflammatory cytokines TGF‐β, IL‐4, and IL‐10 were increased in cells treated with netrin‐1 at 3 and 7 d after implantation. There was no significant difference after 1 d between the three groups, indicating that small‐diameter TEBVs caused inflammation at 1–3 d after implantation. The results also revealed continuous inflammation in control and MRS groups because both proinflammatory and anti‐inflammatory cytokines were high at 14 and 30 d in the two groups and low in netrin‐1‐modified groups (Figure 5D). Taken together, these results indicated that netrin‐1 induced higher levels of anti‐inflammatory cytokines and lower levels of proinflammatory cytokines.

### 2.6 The Inflammation Resolution Microenvironment Provided by Netrin‐1 Promotes EPCs Differentiation, and Small‐Diameter TEBVs Achieve Quicker and Better Endothelialization

**Figure 6:**
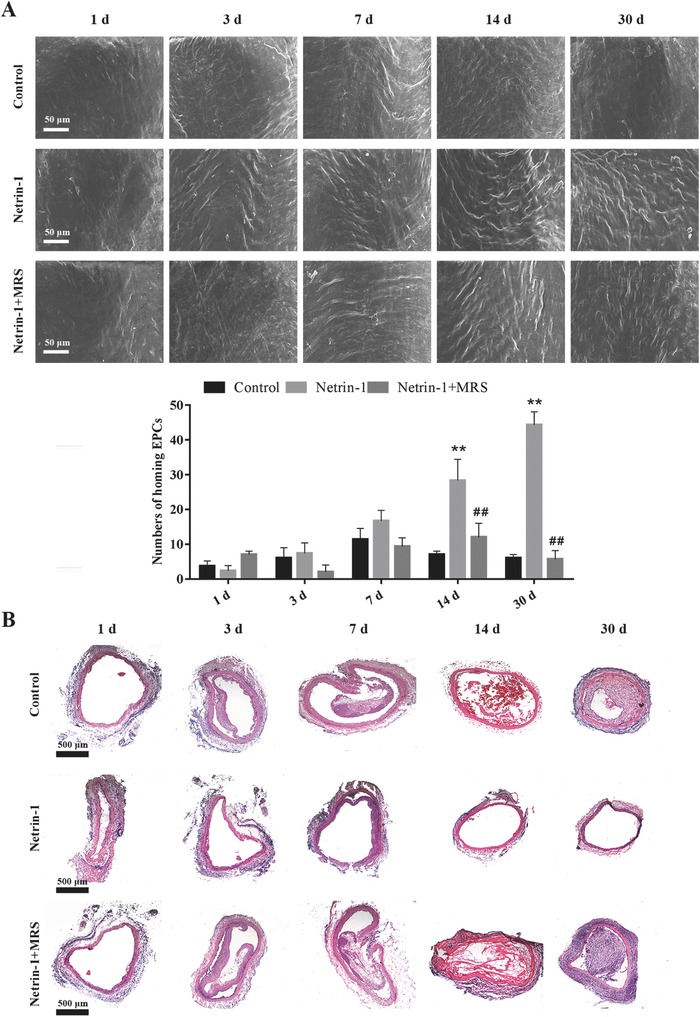
Endothelialization process of small‐diameter TEBVs. A) SEM images of the intimal layer of the different TEBV groups at various time intervals 1, 3, 7, 14, and 30 d. The quantitative comparison of ECs in each group. Results are expressed as mean ± SE, **p < 0.01 versus control, ##*p* < 0.01 versus Netrin‐1 (*n* = 10). B) Representative H&E images corresponding to SEM images in (A). All data are representative of ten independent experiments.

Thirty days after transplantation, more evidence of endothelialization was observed on the intimal layer of small‐diameter TEBVs in netrin‐1 groups compared to the control, as observed by SEM (**Figure** **6**A). The numbers of ECs had no significant difference during the initial 7 d after implantation, but more ECs were prevalent on the intimal layer of small‐diameter TEBVs at 14 d in netrin‐1 compared to the control and MRS groups. Additionally, we found that endothelialization was achieved by day 30 in the netrin‐1 group, while there were few viable cells on the intima of other groups. H&E staining showed similar results and demonstrated that small‐diameter TEBVs kept patent at 30 d in the netrin‐1 group, but there was obvious intimal hyperplasia at 30 d in small‐diameter TEBVs in the control and MRS group (Figure 6B).

In addition, we also found that netrin‐1 could reduce platelet adhesion to the A2b receptor in the acute stage of small‐diameter TEBVs implantation prior to endothelialization. We performed a set of flow‐chamber experiments to determine if netrin‐1 altered the adhesion of platelets to the luminal surface of small‐diameter TEBVs. Significantly fewer platelets were observed on the surface of netrin‐1‐modified small‐diameter TEBVs than on control or A2b‐blocked small‐diameter TEBVs (Figure S4A,B, Supporting Information). Treatment with netrin‐1, but not with netrin‐1 and the A2b‐blocker, significantly increased cyclic Adenosine monophosphate (cAMP) levels in cultured platelets. Therefore, netrin‐1 release caused a decrease in the risk of thrombosis (Figure S4C, Supporting Information).

### 2.7 Netrin‐1‐Modified Small‐Diameter TEBVs Improve Long‐Term Patency

**Figure 7:**
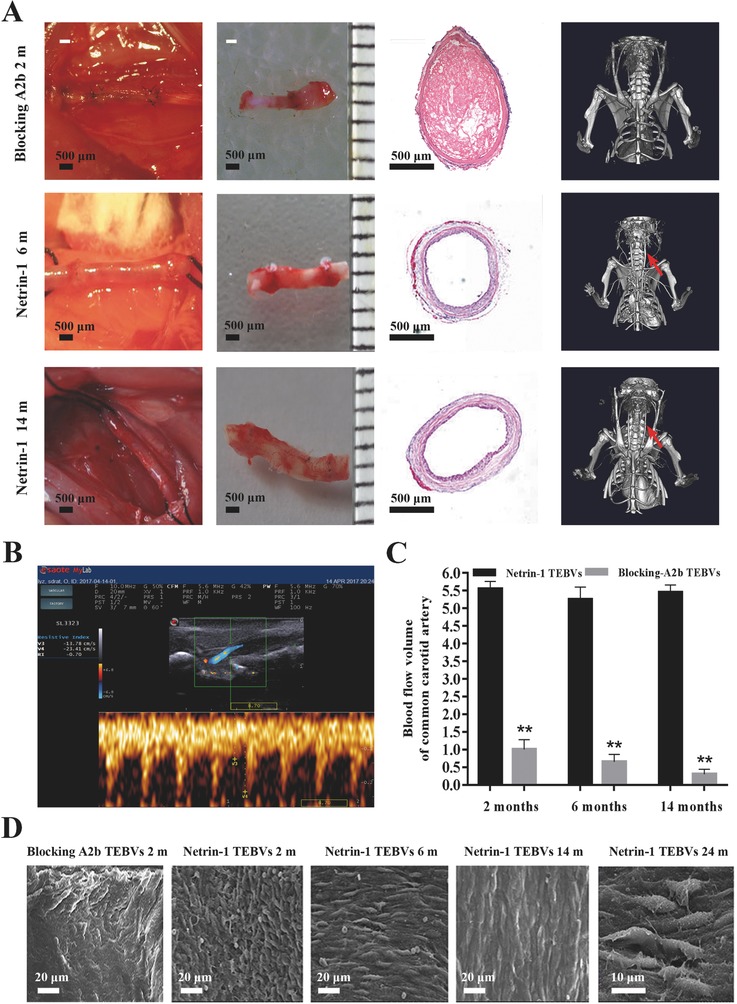
Netrin‐1‐modified small‐diameter TEBVs accomplish endothelialization in time and keep patent for 14 months. A) Dissection, H&E staining, and CTA revealed the characteristics of the small‐diameter TEBVs among different groups at different time points; the red arrows indicate the small‐diameter TEBVs in vivo. B) Laser Doppler ultrasound imaging assessment of netrin‐1‐modified small‐diameter TEBVs transplanted for 14 months and synchronization of pulsation with adjacent host aorta. C) Blood flow volume of small‐diameter TEBVs in different groups were determined by Doppler after being transplanted into rats. *p < 0.05 versus netrin‐1‐modified small‐diameter TEBVs. Values represent the mean ± SD (*n* = 10). D) SEM image of the luminal surface of the small‐diameter TEBVs among different treatment groups at 2, 6, 14, and 24 months. All data are representative of ten independent experiments.

We used the laser Doppler ultrasound imaging and microcomputed tomography angiography (CTA) to assess the blood flow and long‐term patency rate at 2, 6, and 14 months. The results showed that 90% of the A2b‐blocked small‐diameter TEBVs had been obstructed at 2 months after implantation, and the average blood flow rate was 1.01 mL min^−1^, decreasing to 0.32 mL min^−1^ at 14 months. However, the blood flow rate of small‐diameter TEBVs in the netrin‐1 group remained steady at 5.35–5.55 mL min^−1^ up to 14 months. H&E staining also showed that there was no smooth muscle cell (SMC) excessive proliferation and no intimal hyperplasia in netrin‐1‐modified small‐diameter TEBVs at 14 months. CTA results showed netrin‐1‐modified small‐diameter TEBVs still kept patent at 14 months (**Figure** **7**A). Netrin‐1‐modified small‐diameter TEBVs maintained normal pulsation, whereas A2b‐blocked small‐diameter TEBVs became occluded (Videos S1 and S2, Supporting Information). Doppler ultrasound image revealed the high patency of the small‐diameter TEBVs and the blood flow velocity under vascular contraction and relaxation (Figure 7B,C and Video S3, Supporting Information).

SEM images revealed that there were few ECs grown on A2b‐blocked small‐diameter TEBVs, and they were disorganized at 2 months after implantation, whereas the netrin‐1‐modified small‐diameter TEBVs had a greater number of ECs and the majority achieved endothelialization. Unlike the critical intimal hyperplasia present in the A2b‐blocked group, the netrin‐1‐modified small‐diameter TEBVs had favorable endothelization at 6 months and the ECs showed polar growth in the direction of the blood flow. At 14 months after implantation, A2b‐blocked small‐diameter TEBVs were totally implosive, while netrin‐1‐modified small‐diameter TEBVs achieved complete endothelialization and the ECs began to fuse and secrete extracellular matrix (ECM). At 24 months after implantation, we could see that the ECs had successfully grown on the small‐diameter TEBVs, and antenna and intercellular junctions were evident at higher magnifications (Figure 7D).

## 3 Discussion

The effect of inflammation on vascular remodeling and early monocyte recruitment on small‐diameter TEBVs has been studied previously;^28^, ^29^ however, there have been no studies regarding the regulation of inflammation to promote quick endothelialization of small‐diameter TEBVs. First, there is a need for a method to deliver netrin‐1 and control its release through the resistance caused by blood flow that also allows small‐diameter TEBVs to capture EPCs from circulation to promote endothelialization. The results showed that the multilayered collagen‐chitosan nanoparticles containing netrin‐1 successfully bonded to small‐diameter TEBVs, which also enhanced their strength and stabilization due to the ionic bond between the carboxylic acid of collagen and amino group of chitosan.^30^, ^31^ In vivo, the results showed netrin‐1 was released evenly for 30 d.

Exogenous netrin‐1 is able to suppress inflammation by engaging A2b, which may play a role in attenuating neutrophil transmigration.^21^ In this study, the results showed that treating MΦ with netrin‐1 induced CD163 expression and suppressed LPS‐induced CD86 expression. The western blot results further proved that netrin‐1 activates STAT6, PPARγ, and glucocorticoid pathways, known anti‐inflammatory MΦ‐polarizing signaling pathways. MRS inhibited the reprograming effect of netrin‐1, indicating that the A2b receptor is important for the effect of netrin‐1 on MΦ.

Increasing evidence indicates that the microenvironment plays a significant role in vascular disease treatments and EPC survival and function;^32^, ^33^, ^34^, ^35^ In vitro, we observed exosome synthesis and secretion and found that exosomes derived from distinct phenotypes of MΦ rapidly entered and delivered their content to EPCs through an active process. The results revealed that EPCs acquired changes in migratory, proliferative, and tube formation properties in response to their communication with MΦ exosomes. Interestingly, the results also showed that CD86‐exosomes had stronger ability of inducing EPCs migration than CD163‐exosomes, confirming that moderate inflammation can promote EPCs homing mediated by exosomes. In addition, CD163‐exosomes remarkably upregulated the proliferation and tube formation of EPCs, whereas CD86‐exosomes significantly downregulated these properties, which confirmed that intense inflammation discourages the survival and function of EPCs. The expression of Meg3 and lncRNA‐MALAT1 was significantly changed in exosomes of CD163‐MΦ and CD86‐MΦ, which had been shown to play an important role in regulating EC function and vessel growth.^36^, ^37^, ^38^

In vivo, the results showed there was a large number of inflammatory MΦ infiltrating the TEBVs in control group and high expression of proinflammatory cytokines in blood plasma at 3–7 d after implantation. The inflammation then became chronic at 14 d and the small‐diameter TEBVs gradually underwent thrombosis. The MΦ that infiltrated netrin‐1‐modified small‐diameter TEBVs were reprogrammed to express CD163 with a high content of anti‐inflammatory cytokines in the plasma, and then the MΦ evacuated from the sites, inflammation gradually subsided, and small‐diameter TEBVs achieved better endothelialization at 14 and 30 d after implantation. These compelling findings suggest that netrin‐1 is capable of reprogramming MΦ, thereby promoting inflammation resolution of small‐diameter TEBVs.

The SEM and H&E staining results indicated that the netrin‐1‐modified small‐diameter TEBVs maintained optimal conditions for EPCs function and accomplished endothelialization in time at the intimal surface. They had significantly less intimal hyperplasia and more ECs on the intimal surface compared to the control and MRS groups at each time interval. Endothelialization on the vascular cavity surface was significantly increased and there was no sign of thrombosis after 30 d in the netrin‐1 group. The CTA and Doppler ultrasound results showed good long‐term patency of netrin‐1‐modified small‐diameter TEBVs.

In summary, netrin‐1‐modified small‐diameter TEBVs can get early inflammation resolution, which contributes to homing EPCs survival and quick endothelialization. Additionally, patients who need small‐diameter TEBVs often have the disease like diabetes or atherosclerosis,^39^, ^40^ so we need further study the inflammatory condition and resolution ways of small‐diameter TEBVs in these disease state.

## 4 Conclusion

These findings suggest that promoting the resolution of inflammation in time is necessary to induce endothelialization of small‐diameter TEBVs in the stages immediately following implantation and to prevent early thrombosis and problems associated with chronic inflammation. Furthermore, we find that netrin‐1 promotes MΦ reprogramming to induce inflammation resolution and the releasing of exosomes from MΦ to target and regulate EPCs, which improves the long‐term patency rate of small‐diameter TEBVs. In the clinical vascular transplantation, inflammation always induces thrombosis and block of vascular graft. So our study will provide new perspective for vascular graft long‐term survives in host, which may serve as a useful treatment for other inflammatory diseases.

## 5 Experimental Section

Detailed methods are provided in the Supporting Information.

## Conflict of Interest

The authors declare no conflict of interest.

## Supporting information

Supplementary

Supplementary

Supplementary

Supplementary
