# Supplementary material for: Netrin‐1 Promotes Inflammation Resolution to Achieve Endothelialization of Small‐Diameter Tissue Engineering Blood Vessels by Improving Endothelial Progenitor Cells Function In Situ
Source: Adv Sci (Weinh). 2017 Sep 28;4(12):1700278. doi: 10.1002/advs.201700278 (PMC5738088; doi:10.1002/advs.201700278)
Supplement: Supplementary file 1 — Supplementary [file ADVS-4-na-s001.pdf]

Copyright WILEY-VCH Verlag GmbH & Co. KGaA, 69469 Weinheim, Germany, 2017.

ADVANCED SCIENCE
Open Access

Supporting Information

for *Adv. Sci.,* DOI: 10.1002/advs.201700278

Netrin-1 Promotes Inflammation Resolution to Achieve Endothelialization of Small-Diameter Tissue Engineering Blood Vessels by Improving Endothelial Progenitor Cells Function In Situ

Yanzhao Li, Simin Wan, Ge Liu, Wang Cai, Da Huo, Gang Li, Mingcan Yang, Yuxin Wang, Ge Guan, Ning Ding, Feila Liu, Wen Zeng, and Chuhong Zhu**

Supporting Information

Netrin-1 Promotes Inflammation Resolution to Achieve Endothelialization of Tissue Engineering Blood Vessels by Improving Endothelial Progenitor Cell Function in Situ

Yanzhao Li [a], *Simin Wan* [a], *Ge Liu* [a], *Cai Wang* [a], *Da Huo* [a], *Gang Li* [a], *Mingcan Yang* [a], *Yuxin Wang* [a], *Ge Guan* [a], *Ning Ding* [a], *Feila Liu* [a], *Wen Zeng* [a*], *Chuhong Zhu* [a*]

[a] Department of Anatomy, National & Regional Engineering Laboratory of Tissue Engineering, State and Local Joint Engineering Laboratory for Vascular Implants, Key Lab for Biomechanics and Tissue Engineering of Chongqing, State Key Laboratory of Trauma, burn and Combined injury, Third Military Medical University, Chongqing 400038, China

*These authors contributed equally to this work. Correspondence and requests for materials should be addressed to Chuhong Zhu (email: zhuch99@yahoo.com) or Wen Zeng (email: zengw0105@163.com)

1. Experimental section

1.1 MΦ isolation and culture

All procedures performed with animals were reviewed and approved by the Institutional Clinical Experiments Committee and Institutional Review Board Service of the Third Military Medical University, Chongqing, China. SD rats (n=10) were sacrificed by CO_2 inhalation and

cervical dislocation. Peritoneal MΦ were obtained and cultured as previously described. Briefly, we injected rats with sterile phosphate buffer saline (PBS) containing penicillin and streptomycin, gently massaged the belly, then aspirated the fluid, and pelleted and resuspended the cells in red blood cell lysis buffer for 1–2 minutes. After centrifugation, cells were cultured in RPMI medium 1640 containing 10% FCS. Nonadherent cells were removed by washing 2-3 h later, and fresh medium was added.

Firstly, different concentrations of murine recombinant netrin-1 (R&D Systems; Minneapolis, USA) including 50, 100, 200, 250, 300 ng/ml were used to treat MΦ and appropriate concentration was used in the next experiments. For vitro reprogramming experiment, MΦ were stimulated respectively with lipopolysaccharide (LPS; Abcam, Cambridge, MA, USA; 1 μg/ml); netrin-1(250 ng/ml); and LPS, netrin-1, and MRS (Abcam; 100 μM) in three groups for 24–48 h (n=10).

1.2 EPCs isolation and culture

Mononuclear cells were isolated from the peripheral blood of SD rats (n=10) via gradient density centrifugation, seeded onto 24-well plates, and cultured at 37°C and 5% CO_2 in media containing 10% FBS, vascular endothelial growth factor (R&D Systems; 10 ng/ml), basic fibroblast growth factor (R&D Systems; 3 ng/ml), heparin (Abcam; 90 mg/ml), penicillin (Abcam; 100 U/ml), streptomycin (Abcam; 100 U/ml), and fungizone (Abcam; 0.25 mg/ml) for 24 h.[1] Next, the medium was changed to remove non-adherent cells and changed again every other day. Early EPCs were harvested after 7 d of culture, and late EPCs were harvested after 2–3 weeks of culturing.[2]

Spleens from SD rats (n=10) were minced into 1-mm³ fragments, ground into solution, and then sequentially passed through 100- and 40-μm mesh. Red blood cells in the resulting renal cells were lysed using red blood cell lysis buffer, and EPCs were isolated and cultured as procedure for isolating EPCs from peripheral blood (PBL) described above.

1.3 Exosomes purification

The MΦ-exosomes isolation procedure was performed as previously described.[3] Briefly, 2 ml serum-free RPMI medium was used for culturing MΦ in each group. After 48 h, cell culture supernatants were enriched by using a Total Exosome Isolation (from cell culture media) kit (Life Technologies, MA, USA) following the manufacturer's instructions (n=10).

Exosomes were labeled by PKH26 Red Fluorescence Cell Linker Kit or PKH67 Fluorescent Cell Linker Kits (Sigma, St Louis, MO) following the manufacturer's instructions, SD rats (n=10) were injected with PBS and 100 mg PKH26 or PKH67-labeled MΦ exosomes through the tail vein. After 24 h, EPCs were isolated from the spleen or PBL and analyzed by flow cytometry to quantify the proportion of cells that internalized MΦ exosomes.

1.4 Flow cytometry

To quantify the phenotypes of stimulated MΦ in each group, MΦ were stained using the following fluorochrome-labeled antibodies: anti-CD86-APC (BD, New Jersey, USA) and anti-CD206-FITC (BD). After staining for surface markers, MΦ were fixed and analyzed with an Accuri C6 flow cytometer (Becton Dickinson), and the results were analyzed using FlowJo software (Treestar, Inc.) (n=10).

1.5 Immunofluorescence

MΦ were seeded on glass coverslips in 6-well plates. After fixation and permeabilization, MΦ in each group were incubated with the primary antibody mouse-anti-CD86 (BD) overnight at 4°C and stained with secondary antibody Alexa Fluor 568 donkey anti-mouse IgG (Life Technologies) for 60 min at 18-24°C. Next, cells were again incubated with rabbit-anti-CD163 (BD) and Alexa Fluor 488 donkey anti-rabbit IgG (Life Technologies), followed by counter-staining of the nucleus

with Hoechst33342 dye (Sigma). Finally, the cells were imaged with a confocal imaging system (LSM780;Zeiss, GER) (n=10).

After fixation with 4% paraformaldehyde for 1 h, small-diameter TEBVs were cut longitudinally and fixed with double-sided tape on coverslips to expose the intima. Next, MΦ that were able to infiltrate small-diameter TEBVs were immunofluorescently stained as described above. small-diameter TEBVs were transferred to another coverslip and imaged with the confocal imaging system (n=10).

1.6 ELISA

Cell culture supernatants and plasma of rats were used to detect the concentrations of inflammatory factors including IL-1β, IL-6, TNF-α, TGF-β, and IL-10 by ELISA kit (Boster Bioengineering Co. Ltd. China) following the manufacturer's instructions (n=10).

1.7 Immunoblotting

Exosomes and cells were lysed and protein content was assessed using BCA Protein Assay Kit (Biospes, Chongqing, China). Next, protein lysates were separated using SDS-PAGE, and transferred to PVDF membrane (Millipore, Billerica, MA, USA). The membrane was then blocked with 5% non-fat milk, and incubated with rabbit anti-CD63 (Bioss, Beijing, China, 1:200), HSP90 (1:200, Bioss) and TSG101(1:200, Bioss) overnight at 4°C. After washing with PBST, membrane was continued to incubate with HRP linked goat anti-rabbit IgG (1:2000, Cell Signaling Technology, Danvers, MA, USA), and the protein bands were visualized using the automatic imager (Tanon 5200, Shanghai, China) (n=10).

1.8 Co-culture experiments

For confocal microscopy, EPCs ($1x10^4$) were seeded on glass coverslips in the lower chamber of 12-well transwell plates. Absence (Control) or PKH26-stained MΦ ($1x10^6$) were added in the upper

chamber of a 0.4-μm pore membrane insert (Corning Transwell, New York, USA) for 24 h. Nuclei were stained with DAPI (blue) and cells were imaged with the confocal imaging system at various time intervals (n=10).

1.9 Nanoparticle trafficking analysis

Analysis of absolute size distribution of exosomes was performed using nanoparticle tracking analysis (NTA, Nano series, Malvern, UK). After isolation, the exosomes were diluted in 1 ml filtered PBS. Exosomes were measured at 23.75 ± 0.5°C and imaged at 25 frames per second for 60 s. Three detection times were performed for each sample (n=10).

1.10 Transmission electron microscopy (TEM)

For the morphology investigation by TEM, 5μl exosome pellet was placed on formvar carbon-coated 200-mesh copper electron microscopy grids, incubated, and subjected to standard uranyl acetate (Sigma) staining and then allowed to semi-dry at 18-24°C. MΦ were post-fixed with 1% osmium tetroxide (Sigma), progressively dehydrated in a graded ethanol series (30–100%), and embedded in epoxy resin. Thin (1-mm) and ultrathin (70- to 80-nm) sections were cut by a microtome (318423, Reichert Nr.) and placed on copper grids as described previously.[4] Next, the cells and exosomes were observed using a TEM (CM-10, Philips Netherlands). Micrographs were used to quantify the secreting process and diameter of exosomes (n=10).

1.11 Quantitative Reverse Transcription Polymerase Chain Reaction

Exosomal RNA was isolated using TRIzol LS Reagent (Invitrogen, Carlsbad, CA) and then reverse transcription polymerase chain reaction (qRT-PCR) was performed to measure miRNA and lncRNA expression following manufacturer's instruction. Relative expression was normalized to GAPDH (n=10).

1.12 EPCs migration, proliferation, and tube formation assay in vitro

EPCs cultured for 7 d were harvested and seeded into the upper chamber of a 24-well transwell migration insert (8μm; Corning). The lower chamber contained basic medium with or without 100 μg/ml exosomes derived from MΦ stimulated with LPS or netrin-1 in each group. After 12 h, the cells on the lower side were fixed and stained with crystal violet. Light microscopy was immediately used to observe the cell morphology and cell count (n=10).

EPCs cultured for 14 d were plated onto a 96-well plate (Costar, 1×10^4 cells per well) and incubated in each group as described above. The cells were incubated for 72 h and then 0.5 ml of 50 mM EdU (RiboBio Co., Ltd.) was added. After incubation for 6 h, EPCs were detected following the manufacturer's protocol (n=10).

Matrigel (BD) was added to 96-well plates (Corning) at 100μl/well and incubated for 30 min. EPCs were then added to the wells and incubated in each group as described above. After 6, 12, and 24 h incubation, calcein (6.25μg/ml, Sigma) was added and EPCs tube formation was assessed by microscopy. The number of closed loops or meshes, branching points (nodes), tubes and total length of the tubes were calculated and quantified using MacBiophotonics ImageJ software (NIH) (n=10).[5]

1.13 Flow-chamber assessments

Platelets flow-chamber assessments were performed as described previously[6] with the following parameters: width, 16 mm; height, 5 mm; volume flow rate 0.2 ml/s, Q=0.103 ml/s. The circulating fluid (M199 medium enriched with 10% fetal calf serum) had a viscosity of μ=1.3 mPa/s, and the wall shear stress was τ=6Qμ/(wh2)=0.2 Pa. The suspending fluid density was 1.1 g/cm, and the mean linear flow rate was 12.9 mm/s, which produced a Reynolds number of 5.45. Circulation was maintained with a Peri-star Pro pump (WPI, USA) (n=10).

1.14 Small-diameter TEBVs construction and implantation

Under sterile conditions, carotid arteries were harvested from SD rats (200 g), rinsed with saline solution, and digested with 0.05% trypsin (Hyclone, Logan, UT, USA) at 37°C and under 5% CO_2 for 40 minutes to remove the cells. Next, nucleic acids were removed with RNase and DNase to obtain a vascular matrix consisting of collagen and elastic fibers.[7] The vascular matrix was incubated with 4 mg/ml collagen (Kensey Nash, Exton, PA, USA) for 24 h to crosslink a layer of collagen to both the internal and external surfaces of decellularized rat carotid arteries, and then incubated with nanoparticles of emulsified chitosan for 24 hours. Then 5 mM EDC was added for 24 h (to crosslink the collagen to the chitosan), and the vascular matrix material was incubated a second time with 4 mg/ml collagen for 24 h. Netrin-1-modified small-diameter TEBVs were prepared with nanoparticles that contained 500 ng/ml netrin-1 (R&D Systems), A2b-blocked small-diameter TEBVs were prepared with nanoparticles containing netrin-1 (500 ng/ml) and 100 μM MRS (Sigma). Netrin-1 was crosslinked to the matrix material by incubating the small-diameter TEBVs with 2 mg/ml of *N*-succinimidyl 3-(2-pyridyldithio) propionate (SPDP, Pierce).[7-9] small-diameter TEBVs were transplanted into the carotid arteries of SD rats (200 g) after heparinization, anesthesia was provided with 10% chloral hydrate (Sigma), and the animals received intraperitoneal injections of heparin (1 mg/kg, Sigma) each day for 5 d after surgery (n=10).

1.15 Patency evaluation

The rats receiving small-diameter TEBVs transplantation were anesthetized, and the blood flow of the carotid artery was measured using Doppler flow analysis (Transonic System Inc T402).[10] Rats were injected with 1 ml heparin through the tail vein for 5 min, then their chests were opened and intravascular contrast agent iohexol (Tianheng Pharmaceutical Company, Nanoprobes, China)

was injected into the heart to evaluate the patency of the grafts in the micro-CT scanner (Skyscan1176, Bruker micro-CT, Kontich, Belgium).[11] After these tests, the transplanted small-diameter TEBVs were removed and SEM was used to examine intimal surface endothelialization. A portion of small-diameter TEBVs frozen sections were stained with H&E staining to examine patency and intimal hyperplasia (n=10).

1.16 Statistical analysis

Quantified data are reported as mean ± SD, and significance was evaluated using the Student's *t*-test. Evaluations were performed with SPSS 13 software package, and a P value of less than 0.05 was considered significant.

References:

[1] W. Zeng, C. Wen, Y. Wu, L. Li, Z. Zhou, J. Mi, W. Chen, M. Yang, C. Hou, J. Sun, C. Zhu, *BIOMATERIALS* **2012**, *33*, 473.

[2] D. L. Bellinger, D. Lorton, R. W. Hamill, S. Y. Felten, D. L. Felten, *BRAIN BEHAV IMMUN* **1993**, *7*, 191.

[3] R. Wang, P. D. Sun, *PLOS ONE* **2014**, *9*, e91133.

[4] S. Sahoo, E. Klychko, T. Thorne, S. Misener, K. M. Schultz, M. Millay, A. Ito, T. Liu, C. Kamide, H. Agrawal, H. Perlman, G. Qin, R. Kishore, D. W. Losordo, *CIRC RES* **2011**, *109*, 724.

[5] S. Y. Chien, C. Y. Huang, C. H. Tsai, S. W. Wang, Y. M. Lin, C. H. Tang, *Clin Sci (Lond)* **2016**, *130*, 667.

[6] W. Zeng, L. Li, W. Yuan, Y. Wei, J. Mi, J. Sun, C. Wen, W. Zhang, D. Ying, C. Zhu, *BIORHEOLOGY* **2009**, *46*, 21.

[7] C. Zhu, D. Ying, J. Mi, L. Li, W. Zeng, C. Hou, J. Sun, W. Yuan, C. Wen, W. Zhang, *BIOMATERIALS* **2008**, *29*, 2628.

[8] J. Ejiri, N. Inoue, S. Kobayashi, R. Shiraki, K. Otsui, T. Honjo, M. Takahashi, Y. Ohashi, S. Ichikawa, M. Terashima, T. Mori, K. Awano, T. Shinke, J. Shite, K. Hirata, H. Yokozaki, S. Kawashima, M. Yokoyama, *CIRCULATION* **2005**, *112*, 2114.

[9] W. Zeng, W. Yuan, L. Li, J. Mi, S. Xu, C. Wen, Z. Zhou, J. Xiong, J. Sun, D. Ying, M. Yang, X. Li, C. Zhu, *BIOMATERIALS* **2010**, *31*, 1636.

[10] J. Andersson, R. Larsson, R. Richter, K. N. Ekdahl, B. Nilsson, *BIOMATERIALS* **2001**, *22*, 2435.

[11] G. Liu, L. Li, D. Huo, Y. Li, Y. Wu, L. Zeng, P. Cheng, M. Xing, W. Zeng, C. Zhu, *BIOMATERIALS* **2017**, *127*, 117.

2. Supplemental table and figures

Table 1. Primer sequences, annealing temperature, and product sizes for quantitative RT-PCR

Target gene/abbreviation	Primer sequence forward/reverse	Annealing temperature (°C)	Size of products (bp)
MALAT1	F:5'TAGAGGGTGGGCTTTTGTTG 3'<br>R:5' AGGCTGGTTATGACTCAGATGGT3'	60	91
Lnc-COX2	F:5'GGCGTGATTTTGCCAGATG 3'<br>R:5'GCCCGTGACTATGTAACAAACC3'	60	166
Meg3	F:5' CCTGGGAGAATCTGGTGGTA 3'<br>R:5' CCTCTTGGCCCTTCTTACTTC 3'	60	133
Ang362	F:5' TGGGAGTAAGACCTGTTAAACC 3'<br>R:5' GCTGATTACGAAAGACAGGATC 3'	60	179
Tie-1ASlncRNA	F:5'TACCGCGGGGACATCTAAAGACAC3'<br>F:5'ATTGGATCCCTTGGTGGCCCAGAAAAC3'	60	200
GAPDH	F:5'GGGAAACTGTGGCGTGAT3'<br>R:5'GAGTGGGTGTCGCTGTTGA3'	60	299

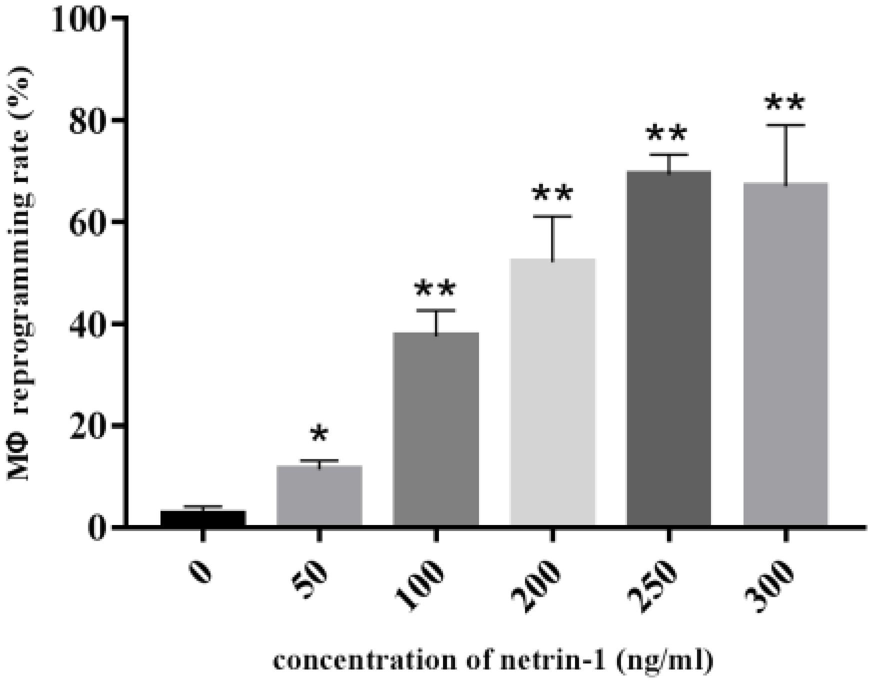

Figure. S1 The concentration of netrin-1 affects the reprogramming rate of MΦ. MΦ extracted from peritoneal fluid were treated by LPS to transform into phenotype of CD86, and different concentrations of netrin-1 were used to treat MΦ. The phenotypes of MΦ were determined by flow cytometry and the reprogramming rate was quantified. Results are expressed as mean ± SE, *p < 0.05, **p < 0.01 versus control.

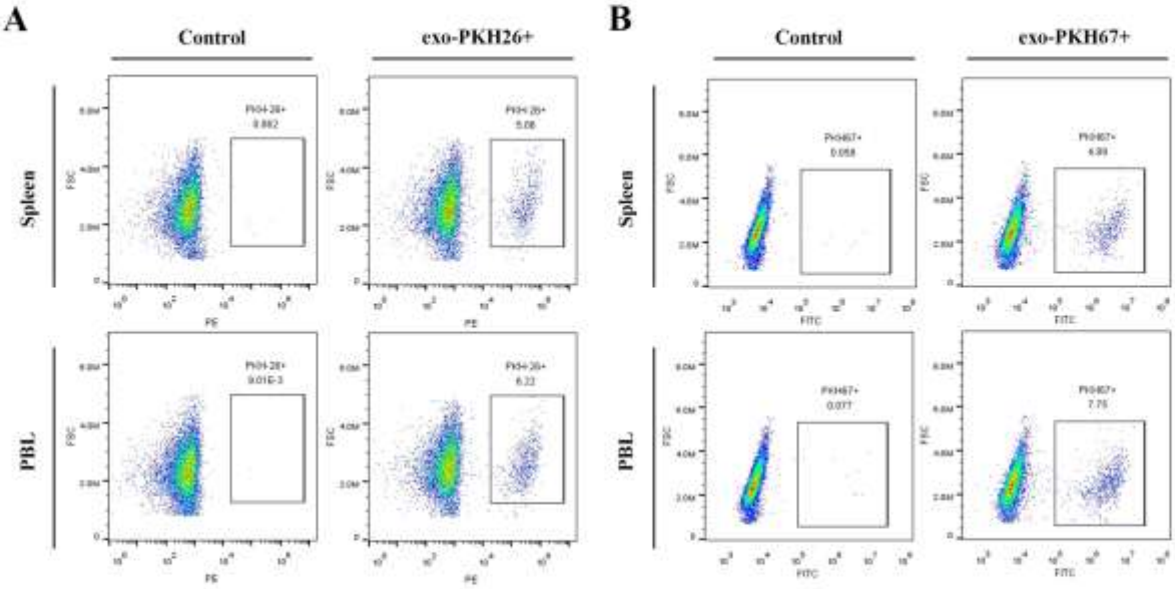

Figure. S2 MΦ-secreted exosomes enter endothelial progenitor cells in vivo. (A) The proportion of PBL EPCs that internalized MΦ exosomes (PKH26+). (B) The proportion of spleen EPCs that internalized MΦ exosomes (PKH67+) (representative of 10 animals).

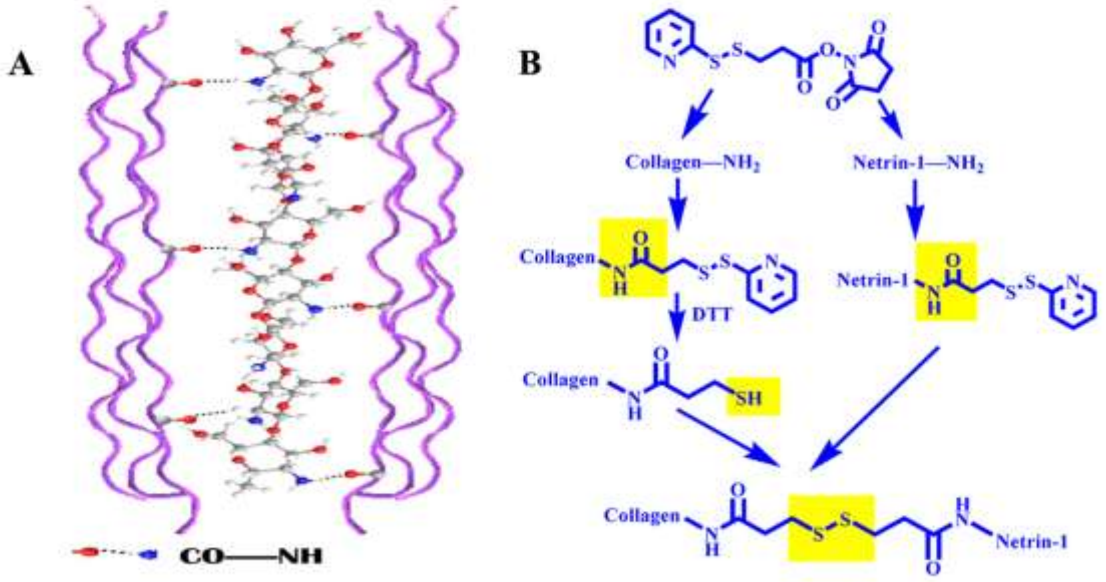

Figure. S3 Link between collagen and chitosan nanoparticles or collagen and netrin-1. (A) Collagen polypeptide chains linked with chitosan nanoparticles through an ionic interaction between the carboxylic acid and amino group. (B) Collagen was coupled with netrin-1 by SPDP.

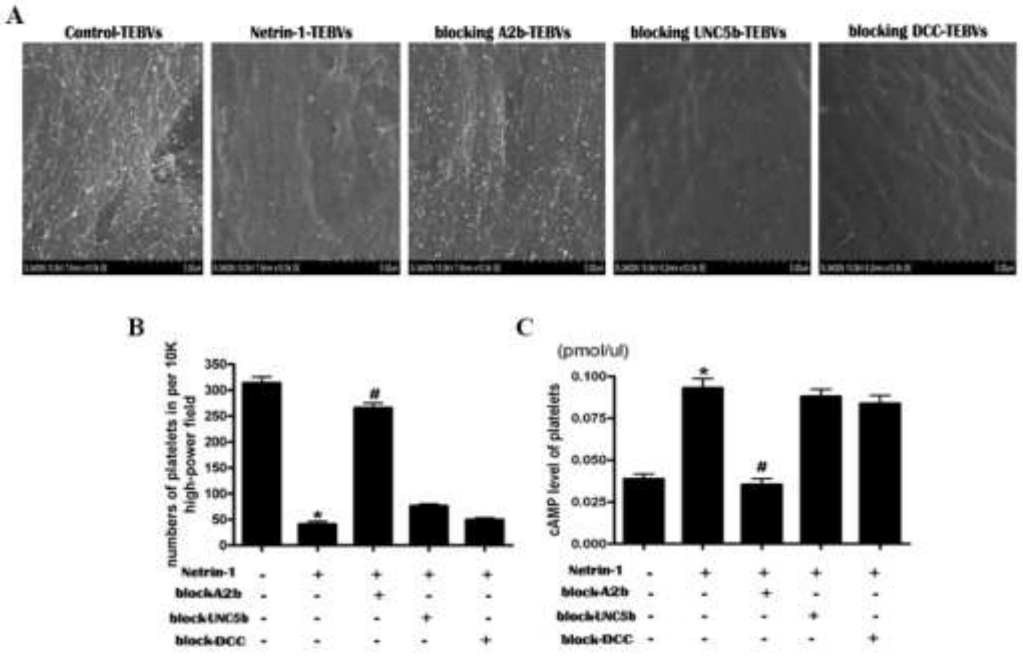

Figure. S4 Netrin-1 reduces platelet adhesion to small-diameter TEBVs. (A) SEM image showing the adherence of platelets on the luminal surface of the different small-diameter TEBVs groups under the flow chamber. (B) The number of platelets adhering to the luminal surface of different small-diameter TEBVs groups was determined. (C) cAMP levels of platelets under different treatments. (D) Aggregation of platelets (red) under different treatments. *p<0.05 (n=10) versus the control group, #p<0.05 (n=10) versus the netrin-1 modified small-diameter TEBVs. Values represent the mean ± SE.

3. Supplemental Video Legends

Video S1 A2b-blocked small-diameter TEBVs became occluded at 2 months after transplantation.

Video S2 Netrin-1 modified small-diameter TEBVs remained patency for 14 months.

Video S3 Doppler vascular ultrasound revealed the patency of netrin-1-modified small-diameter TEBVs at 14 months after transplantation.
